# The Pharmacopœia of the United States of America

**Published:** 1883-01

**Authors:** 


*The Pharmacopœia of The United States of America.*—Sixth Decennial Revision. By Authority of the National Convention for Revising the Pharmacopœia, held at Washington, A. D. 1880. Publishers: William Wood & Co. New York, 1882,

The fifth edition of this Pharmacopœia appeared in 1873, hence the present edition contains much more valuable information, as it is revised after an interval of nearly ten years. The revised nomenclature on the basis of certain general principles, appears in the present edition. The well known Publishers have presented the work in an admirable style, with large and clear type, and in every respect attractive and useful.

